# Arthroscopic reconstruction of anterior cruciate ligaments with allograft: single-tunnel single-bundle versus single-tunnel double-bundle techniques

**DOI:** 10.1186/s10195-022-00649-4

**Published:** 2022-06-27

**Authors:** Zhenhan Deng, Yizi Zheng, Zhiqin Deng, Changwei Lu, Yihua Wu, Kang Chen, Zicai Fu, Hui Zhang, Wei Lu, Weimin Zhu

**Affiliations:** 1 Department of Sports Medicine, The First Affiliated Hospital of Shenzhen University, Shenzhen Second People’s Hospital, Shenzhen, 518035 Guangdong China; 2 Guangxi University of Chinese Medicine, Nanning, 530200 Guangxi China; 3 Hand and Foot Surgery Department, The First Affiliated Hospital of Shenzhen University, Shenzhen Second People’s Hospital, Shenzhen, 518035 Guangdong China; 4 Department of Spine and Joint Orthopedics, The People’s Hospital of Hechi, Hechi, 547000 Guangxi China

**Keywords:** Arthroscopy, Anterior cruciate ligament, Double-bundle reconstruction, Anterior tibialis tendon, Allograft

## Abstract

**Purpose:**

To compare the clinical results of anterior cruciate ligament (ACL) reconstruction using the single-tunnel single-bundle (STSB) technique versus the single-tunnel double-bundle (STDB) technique.

**Methods:**

This was a retrospective, single-center, single-surgeon study based on data collected from March 2012 to June 2013. According to our inclusion/exclusion criteria, a total of 78 patients (64 males, 14 females; mean age, 25.1 years) who underwent arthroscopic ACL reconstruction with anterior tibialis tendon allografts through either the STSB technique (36 cases) or the STDB technique (42 cases) in our department were recruited. The International Knee Documentation Committee (IKDC), Lysholm, and Tegner scores were used to evaluate the subjective function of the knee joint during the postoperative follow-up. The Lachman test and pivot shift test were used to objectively assess the stability of the knee.

**Results:**

The average follow-up duration was 24.9 ± 1.8 months in the STSB group and 24.6 ± 1.7 months in the STDB group (*P* > 0.05). Patients in both groups recovered to the preoperative sports level with few complications. The postoperative Lysholm score (86.1 ± 7.5 vs. 47.7 ± 9.0 in the STSB group; 87.0 ± 7.1 vs. 48.2 ± 8.3 in the STDB group), IKDC score (87.8 ± 7.2 vs. 49.3 ± 6.1 in the STSB group; 88.7 ± 6.6 vs. 49.8 ± 6.3 in the STDB group), Tegner score (6.5 ± 1.3 vs. 2.5 ± 1.3 in the STSB group; 6.6 ± 1.2 vs. 2.6 ± 1.2 in the STDB group), Lachman test positive rate (8.3% vs. 89.9% in the STSB group; 7.1% vs. 85.7% in the STDB group), and pivot shift test positive rate (27.8% vs. 63.9% in the STSB group; 7.1% vs. 69.0% in the STDB group) were significantly improved compared to the preoperative status in both groups (*P* < 0.05). However, no statistically significant difference was observed between the two groups at the final follow-up (*P* > 0.05), except for the pivot shift test positive rate in the STDB group versus the STSB group (7.1% vs. 27.8%, *P *< 0.05).

**Conclusions:**

The STDB technique achieved a satisfactory clinical outcome with better rotational stability compared to the traditional STSB technique and therefore provided an effective option for ACL reconstruction.

**Level of evidence:**

Case series, Level IV.

## Introduction

The anterior cruciate ligament (ACL) is the main stabilizing structure of the knee; it resists anterior translation and maintains forward and rotational stability [1]. The ACL is generally considered to include two functional bundles, i.e., the anteromedial (AM) and posterolateral (PL) bundles, each having a unique function [2, 3]. The AM bundle and PL bundle provide anteroposterior and rotational stability to the knee joint [4]. At present, single-tunnel single-bundle (STSB) and double-tunnel double-bundle (DTDB) reconstructions are two of the main ACL surgical procedures available in clinical practice [5, 6]. However, DTDB is technically difficult to perform for surgeons and more traumatic for patients compared to STSB. It requires a longer operative time, involves an increased risk of graft impingement and lateral femoral condyle and bone bridge fractures, and is challenging in revision surgery [7].

Taking these factors into account, some surgeons have explored single-tunnel double-bundle (STDB) reconstruction with the goal of restoring the anatomical double-bundle (DB) structure of the ACL within a single tunnel [8–11]. This technique overcomes the shortcomings of both DTDB and STSB reconstruction (i.e., restoring the anteroposterior and rotational stability while avoiding the drawbacks of DTDB reconstruction). Previous publications also reported a DB ACL reconstruction technique with one femoral and two tibial tunnels, which achieved good rotational stability in cadaveric knees [12, 13]. Duncan et al.’s study of fresh-frozen porcine knees with 40 samples concluded that fixation of the ACL with a double-tunnel technique on the tibial side had a biomechanical advantage with no potential deleterious side effects [14]. Another study found that the three-tunnel DB (with two femoral tunnels and a single tibial tunnel or with a single femoral tunnel and two tibial tunnels) could better restore intact knee biomechanics than single-bundle ACL reconstruction in a porcine model [15].

However, no patient outcome data have been reported yet. Whether the STDB ACL technique is able to restore knee joint stability and whether the patients can return to sports remain unknown. Therefore, clinical data for patients with an ACL rupture undergoing arthroscopic STSB (one femoral and tibial tunnel with one bundle) or STDB (one femoral and tibial tunnel with two bundles) reconstruction in our department were reviewed to compare the two surgical procedures in terms of the functional outcome, joint stability, complications, and side effects. The hypothesis of this study is that the STDB technique is an effective procedure to restore the knee stability of ACL injury patients and is able to achieve a better clinical outcome without introducing more complications than the traditional STSB technique.

## Methods

### Inclusion and exclusion criteria

The inclusion criteria were: (1) patients had subjective instability, and abnormal knee laxities were confirmed by the Lachman test and pivot shift test; (2) ACL rupture was confirmed by magnetic resonance imaging (MRI); (3) 18 years of age or over; (4) unilateral primary ACL injury; (5) patients had no or minimal osteochondral degeneration on radiographic examination; (6) patients underwent arthroscopic STDB or STSB ACL reconstruction with anterior tibialis tendon allografts.

The exclusion criteria were: (1) damage to multiple ligaments or injury of the articular cartilage; (2) radiographic evidence of Kellgren–Lawrence grade 3 or 4 osteoarthritis (OA) and/or severe osteoporosis; (3) bilateral ACL injuries; (4) partial ACL rupture; (5) concomitant total or subtotal meniscectomy; (6) young patients with unclosed growth plates.

### Patient information

**Fig. 1:**
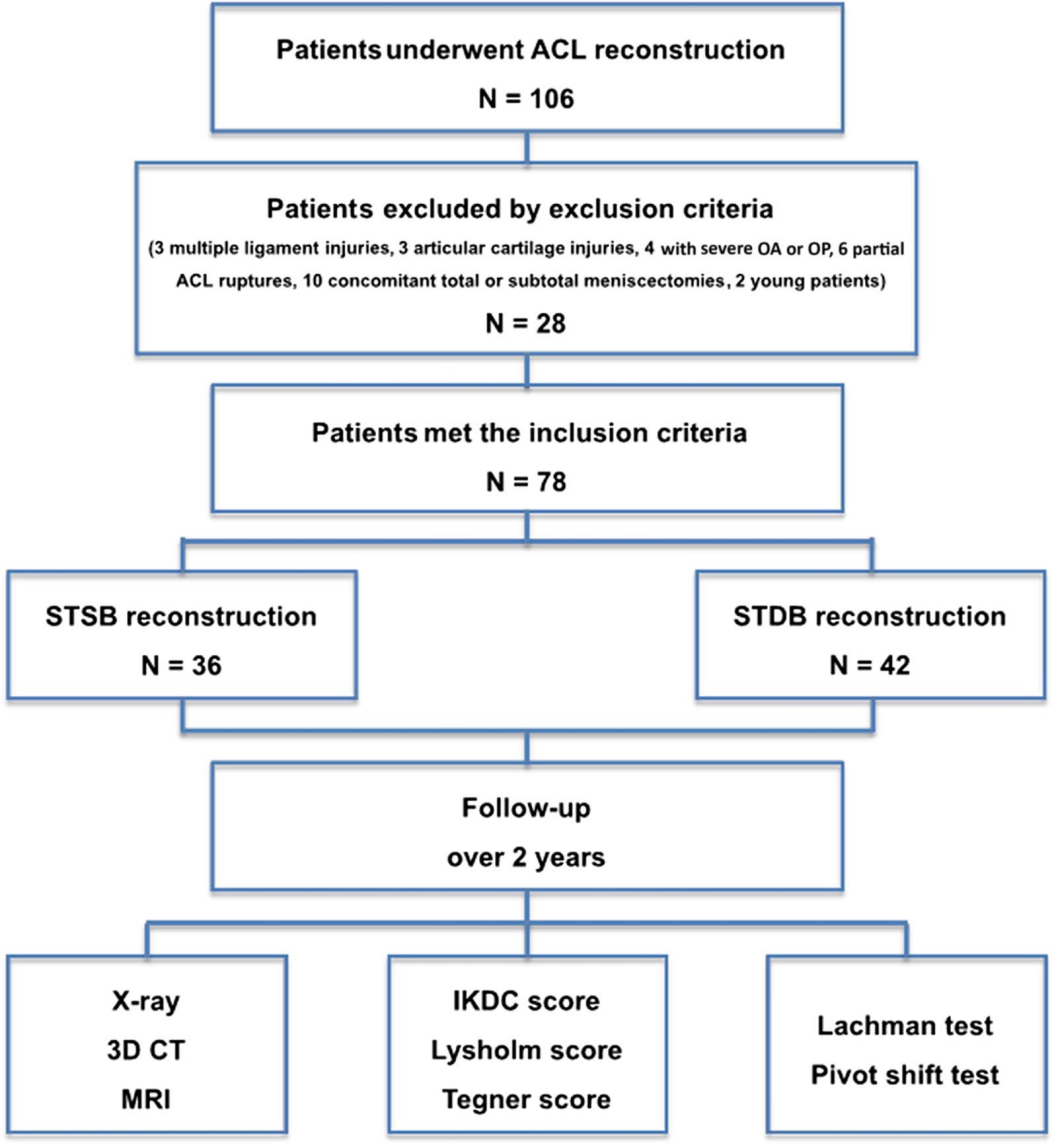
Flow diagram of the study design

This retrospective study was carried out upon receiving approval from our institution’s ethical review board. Overall, 78 patients who visited our department from March 2012 to June 2013 met our inclusion criteria and were recruited for this study. The duration from injury to surgery ranged from 3 days to 12 months. There were 60 patients with a meniscus injury, for whom the menisci were sutured, shaped, or resected according to the type of injury. All of the surgeries were performed by the same senior surgeon, with either STSB reconstruction (*N * = 36) or STDB reconstruction (*N* = 42) performed, which was randomized with closed envelopes. A flowchart of the patient selection process is presented in Fig. 1.

### Allograft preparation

The anterior tibialis tendon allografts (Bone Tissue Engineering Library, Shanxi, China) were prepared on a back table after thawing in 37 °C normal saline. The length of the tendon (24–30 cm) was measured and doubled on itself; then the ends of the tendon were whip-stitched for about 35 mm with No. 2 Fiberwire suture (Arthrex). In the STSB group, the allograft was folded and weaved into a single bundle with a length of over 7 cm and a diameter of 8–9 mm. In the STDB group, the allografts were separated into AM and PL bundles. The AM bundle was over 6.5 cm in length and 6–7 mm in diameter, while the PL bundle was over 5.5 cm in length and 5–6 mm in diameter. Then the graft was clamped at either end on the preparation board with 10 lb of tension. The tendon allograft was kept moist until implantation.

### Anesthesia and exposure

The surgeries were performed on patients in a supine position, with the affected knee flexing at an angle of 90° to allow the lower leg to naturally droop beside the bed. Epidural anesthesia was administered. A tourniquet was applied around the upper thigh. A routine external anterior approach under arthroscopy was performed in order to confirm the diagnosis of a torn ACL.

### STSB ACL reconstruction

**Fig. 2:**
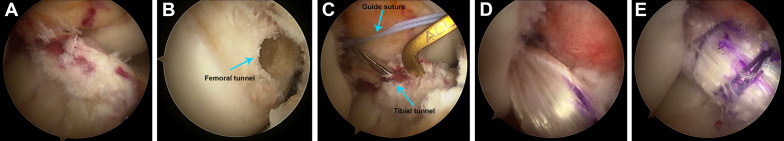
Surgical procedures for STSB ACL reconstruction under arthroscopy. **A** Diagnosis of ACL rupture under arthroscopy. **B** Femoral tunnel. **C** Tibial tunnel. **D** Graft was pulled into the femoral tunnel. **E** Reconstructed ACL

The knee was examined by arthroscopy following conventional procedures to confirm the diagnosis (Fig. 2A). In accordance with our previous publication [2], both the lateral intercondylar ridge and the lateral bifurcate ridge were important bony landmarks for the femoral attachments of the ACL. The femoral tunnel, which should not surpass the lateral intercondylar ridge, was created in the center of the lateral bifurcate ridge. A K-wire was placed into the lateral femoral condyle at the 1:30 or 10:30 position through the AM portal using a freehand technique at 120° of knee flexion (Fig. 2B). Using the inserted K-wire as the reference, a femoral tunnel was reamed to the lateral cortex of the distal femur using a 4.5 mm EndoButton drill. A 30 mm femoral socket that matched the prepared graft diameter was then created using a cannulated reamer. The tibial tunnel was placed at the center of the ACL remnant through the AM surface of the tibia at the level of the tibial tubercle using a tibial guide (Smith & Nephew Acufex) (Fig. 2C). The graft was first introduced into the tibial tunnel with a guide wire and then pulled directly into the femoral tunnel and fixed on the femoral side by flipping over the EndoButton (Smith & Nephew) (Fig. 2D, E). The tibial side was fixed using a hydroxyapatite interference screw (DePuy Mitek) with a diameter 1 mm larger than the graft at 3° of knee flexion under 40 N of initial tension.

### STDB ACL reconstruction

**Fig. 3:**
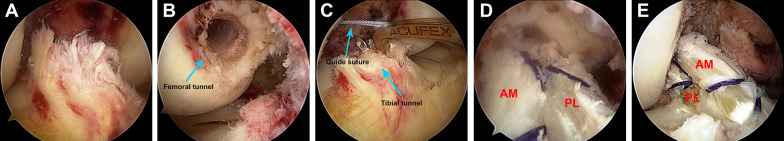
Surgical procedures for STDB ACL reconstruction under arthroscopy. **A** Diagnosis of ACL rupture under arthroscopy. **B** Femoral tunnel. **C** Tibial tunnel. **D** Grafts were pulled into the femoral tunnel. **E** Reconstructed ACL

**Fig. 4:**
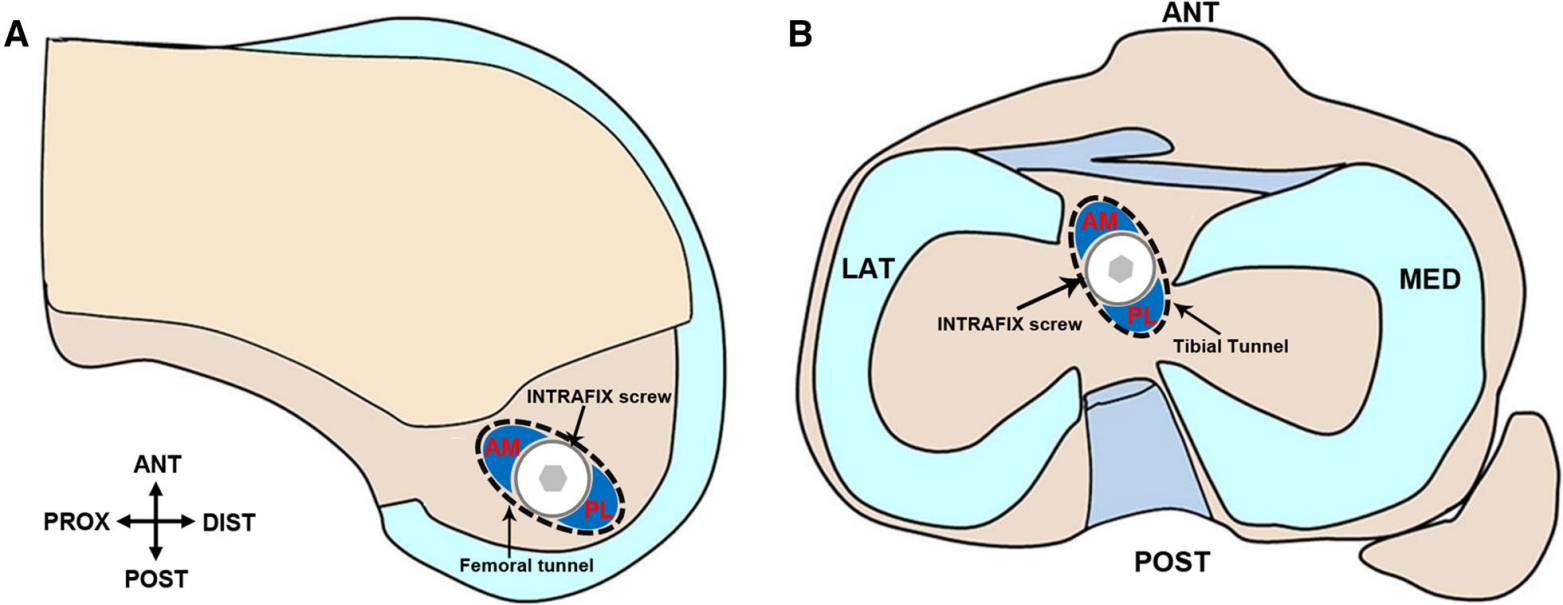
Illustrative surgical diagrams for STDB ACL reconstruction. **A** Femoral insertion in medial surface of the lateral femur condyle. **B** Tibial insertions (AM tunnel and PL tunnel) into the tibial plateau. *ANT* anterior, *DIST* distal, *LAT* lateral, *MED* medial, *POST* posterior, *PROX* proximal

After confirming the ACL rupture (Fig. 3A), both the femoral and tibial tunnels were created using a method similar to the STSB technique (Fig. 3B, C). The AM and PL bundles were looped over a single strand of suture, and a graft-positioning tool was used to achieve the desired position for each bundle. The graft was placed in the fork of the positioning tool with one bundle on either side of the fork. The single strand of the suture over which the graft was looped was passed through the femoral tunnel until it was out of the lateral thigh, and this suture was used to pull the graft into the tunnel. The graft-positioning tool was advanced through the tibial tunnel until it reached the aperture of the femoral tunnel. At this point, the AM and PL bundles were rotated by rotating the positioning tool to achieve their desired positions before they were advanced into the femoral tunnel (Fig. 3D, E). A femoral INTRAFIX screw (DePuy Mitek) was driven between the strands to separate the two bundles within the single tunnel. For the tibial tunnel fixation, the two bundles were placed in opposite quadrants of the sheath at their anatomical insertion sites on the tibial plateau using the tibial INTRAFIX system (DePuy Mitek). While the graft was secured, 40 N of graft tension were applied by an interference screw at full extension. Illustrative surgical diagrams are presented in Fig. 4.

### Postoperative treatment and rehabilitation

Cefoxitin 1 g bid was administered during the first 48 h postoperatively to prevent infection in all the patients. The affected limb was wrapped in cotton pad for 72 h. Three-dimensional computed tomography (3D CT) was performed immediately after surgery to evaluate the bone tunnel and fixation, and MRI was applied to check the ligament healing at the last postsurgical follow-up.

The same postoperative rehabilitation plan was executed in both groups. The affected limb was immobilized with adjustable support. The patients were allowed to walk with crutches while being protected properly by knee braces on the second day after surgery. They were encouraged to flex their knees from 0 to 90° within 2–4 weeks and further to 120° within 6–8 weeks. However, they were instructed not to flex the knee over 120° in the first 3 months postoperatively. The braces were worn for at least 2 months. The patients were allowed to swim and ride a bicycle 6 months after surgery, begin jogging 10 months after surgery, and participate in strenuous exercises 18 months after surgery [16].

### Outcome measures

Clinical outcome was assessed based on the International Knee Documentation Committee (IKDC), Lysholm, and Tegner scores and physical examinations performed both before surgery and at the last follow-up for all the patients.

### Statistical analysis

Data were expressed as the mean ± standard deviation (SD) and analyzed by SPSS 18.0 software (SPSS Inc., Chicago, IL, USA). The independent-samples *t*-test and *χ*^2^ test were performed on the general data from the patients. Preoperative and postoperative IKDC, Lysholm, and Tegner scores and KT-3000 measurements were tested for Mann–Whitney *U* rank. Fisher’s exact test was used for the Lachman test and pivot shift test. *P * < 0.05 was considered statistically significant.

## Results

### Demographic characteristics and follow-up

The demographic characteristics of the 78 patients included are listed in Table 1. There was no statistical difference between the two groups in terms of gender, age, affected side, injury time interval, and follow-up duration.

**Table 1:** General information on the patients

Group	Number	Gender		Age (years)	Side		Injury time (months)	Follow-up (months)
		Male	Female		Left	Right		
STSB group	36	30	6	25.6 ± 4.9	24	12	3.7 ± 2.8	24.9 ± 1.8
STDB group	42	34	8	24.6 ± 4.7	26	16	3.5 ± 2.7	24.6 ± 1.7
*W*/*χ*^2^	–	*χ*^2^ = 0.0		*W* = 663.0	*χ*^2^ = 0.0		*W* = *725.5*	*W* = 687.0
*P*	–	1.00		0.35	0.93		0.76	0.46

Note: the independent-samples *t*-test and *χ*^2^ test were used

Two patients in each group showed extension limitations preoperatively. Loose bodies were detected in the intercondylar fossa and were removed during arthroscopic examination. Postoperatively, all patients showed full extension of the knees. The average follow-up duration was 24.9 ± 1.8 months in the STSB group and 24.6 ± 1.7 months in the STDB group.

### Clinical outcomes

**Fig. 5:**
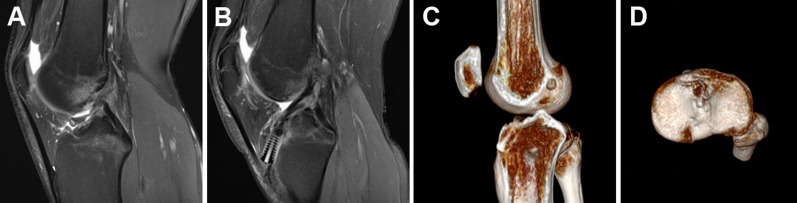
Radiological examination of STSB ACL reconstruction. **A** Preoperative MRI showed ACL rupture. **B** MRI at the last follow-up showed complete graft healing. **C** 3D CT showed the femoral socket and the tibial tunnel. **D** 3D CT showed the tibial socket

**Fig. 6:**
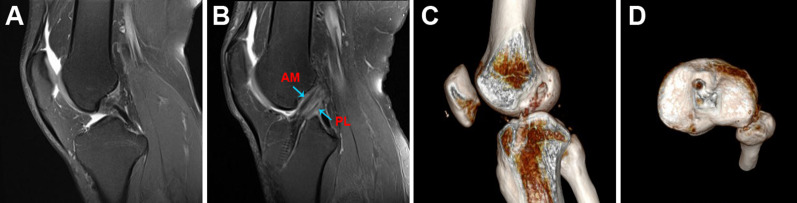
Radiological examination of STDB ACL reconstruction. **A** Preoperative MRI showed ACL rupture. **B** MRI at the last follow-up showed complete graft healing. **C** 3D CT showed the femoral socket and the tibial tunnel. **D** 3D CT showed the tibial socket

Postoperative 3D CT showed accurate bone tunnels and properly positioned screws in both groups, and the low signal intensity of the ACL graft in the T2-weighted MRI at the last follow-up suggested graft maturation in both groups (Figs. 5, 6). No radiograph indicated joint space narrowing or degenerative change at the last follow-up.

All the patients had recovered their preoperative activity level at the last follow-up. The IKDC, Lysholm, and Tegner scores at the last follow-up were significantly improved in both groups compared to their preoperative status (*P* < 0.01), but there was no significant difference between the two groups preoperatively and at the last follow-up (Table 2).

**Table 2:** IKDC, Lysholm, and Tegner scores in both groups preoperatively and at the last follow-up

Group	Number	IKDC score		*P* value	Lysholm score		*P* value	Tegner score		*P* value
		Preoperative	Follow-up		Preoperative	Follow-up		Preoperative	Follow-up	
STSB	36	49.3 ± 6.1	87.8 ± 7.2	< 0.01	47.7 ± 9.0	86.1 ± 7.5	< 0.01	2.5 ± 1.3	6.5 ± 1.3	< 0.01
STDB	42	49.8 ± 6.3	88.7 ± 6.6	< 0.01	48.2 ± 8.3	87.0 ± 7.1	< 0.01	2.6 ± 1.2	6.6 ± 1.2	< 0.01
*P* value	–	0.75	0.57	–	0.81	0.59	–	0.68	0.81	–

The Mann–Whitney *U* rank test was used

Preoperatively, the Lachman test was positive in 32 patients (positive rate 88.9%) in the STSB group and 36 patients (positive rate 85.7%) in the STDB group (*P* = 0.74), and the pivot shift test was positive in 23 patients (positive rate 63.9%) in the STSB group and 29 patients (positive rate 69.0%) in the STDB groups (*P* = 0.63). The positive rates were significantly decreased at the last follow-up when compared to the preoperative status (*P* < 0.01). No significant difference was found in the Lachman test positive rate at the final follow-up between the two groups (*P* > 0.05). However, the pivot shift test rate was significantly lower in the STDB group than in the STSB group at the final follow-up (*P* = 0.01, Table 3).

**Table 3:** Comparison of the number of positive Lachman tests and pivot shift tests obtained preoperatively and at the last follow-up in both groups

Groups	Total number	Lachman test positive rate (*n*)		*P* value	Pivot shift test positive rate (*n*)		*P* value
		Preoperative	Follow-up		Preoperative	Follow-up	
STSB	36	32	3	< 0.01	23	10	< 0.01
STDB	42	36	3	< 0.01	29	3	< 0.01
*P* value	–	0.74	1.00	–	0.63	0.01	–

Fisher’s exact test was used

### Complications

There was no statistically significant difference in complications between the two groups (each group reported one case of infection who recovered after systematic anti-infection therapy). There were two cases in the STSB group and three cases in the STDB group who developed occasional pain and residual subjective joint instability. No stiffness, rejection reaction, graft failure, or deep venous thrombosis of the lower extremities was found in either group.

## Discussion

Our results across over 2 years of follow-up indicated that the pivot shift test positive rate in the STDB group was significantly lower than that in the STSB group but that there was no significant difference in IKDC, Lysholm, and Tegner scores between the two groups at the final follow-up. Both techniques were found to improve knee joint function significantly compared with the preoperative status.

The argument regarding which technique is better for ACL reconstruction—STSB or DTDB—continues to this day [17, 18]. It has been proven that each of the two bundles of the ACL plays an irreplaceable role. Zantop et al. reported that the tibia shifted forward more significantly after transecting the AM bundle in the knee at 60° and 90° flexion and after transecting the PL bundle in the knee at 30° flexion [3]. Additionally, when flexing at 0° and 30°, the knee revolved more significantly after transecting the PL bundle than after transecting the AM bundle and in the normal situation. Several studies have found that there was no difference in the postoperative functional assessment and complications rate, but STSB reconstruction was inferior at restoring knee stability, particularly rotational stability [19, 20]. Järvelä et al. indicated that there was no significant difference in anterior stability between the two procedures, but DTDB gave significantly superior rotational stability [19], while Siebold et al. reported that DTDB led to superior anterior and rotational stability compared to the STSB procedure [20]. STDB reconstruction, as an alternative treatment option for restoring normal anatomic structures and biomechanical properties [21], involves reconstructing two bundles (AM and PL) with different functions by separating the two bundles of ligaments in the tunnel. This technique is characterized by a simple operation, an ability to simulate DTDB anatomic properties, and excellent anterior and rotational stability, and it can shorten the surgical time and decrease the difficulty and risk of the DBDT pattern [22, 23]. Gadikota et al. measured the biomechanical properties of a cadaveric knee specimen in the action of forward loading and found that STDB was more equivalent to a normal ACL in terms of its biomechanical properties compared to STSB reconstruction [24].

In this study, clinical outcomes, Tegner and Lysholm scores, and IKDC grades were compared between the STSB and STDB reconstruction procedures. Although no significant difference between the procedures in clinical functional scores and Lachman test positive rates was noted, the STDB technique showed a significantly lower pivot shift test positive rate than STSB at the last follow-up, indicating that STDB achieved better rotational stability. Ping et al. compared the therapeutic effects of bioabsorbable interference screws with EndoButtons fixation of the grafts in the treatment of ACL rupture through the STDB technique. No significant difference was found in Lysholm, IKDC, and Larson scores at the last follow-up [12]. In a later cadaveric study by those authors, biomechanical analysis was performed to compare STDB with STSB reconstruction using both methods. The results indicated that similar anterior–posterior stability was achieved using the two techniques, while STDB exhibited better rotational stability tested at 30° and 45° of knee flexion than STSB, which was consistent with our findings [13]. In Meuffels et al.’s study of fresh-frozen porcine knees, no significant difference in maximum failure load was found between the two techniques. However, the stiffness of the tibial tunnel complex was significantly higher in the STDB group [14]. Therefore, our results further strengthened the evidence supporting the application of this new technique in clinical practice.

Despite the merits shown, this study presents some limitations as well. Firstly, as it is a retrospective study, more prospective research, including randomized controlled trials, should be performed to provide further evidence. Secondly, the sample size was relatively small and there was a relatively short follow-up duration. More cases will be included and longer-term investigations will be conducted in the future. Moreover, previous cadaveric studies reported that the STDB technique might achieve better rotational stability [12, 13]. However, no quantitative data on the difference between the two methods could be collected from the patients. Only the clinical physical examination and the patient’s objective feeling of stability were measured in this study. Last but not least, the clinical outcome was not compared between STDB and DTDB, which points to a future direction for our research.

## Conclusions

The stability and function of the knee joint can be restored well using either STSB or STDB ACL reconstruction with allograft. The STDB technique showed superior rotational stability at the final follow-up, accompanied by a satisfactory short-term curative effect.

## Data Availability

All relevant data for the research are included in the manuscript.

## Abbreviations

3D CT: Three-dimensional computed tomography
ACL: Anterior cruciate ligament
AM: Anteromedial
DB: Double-bundle
DTDB: Double-tunnel double-bundle
IKDC: International Knee Documentation Committee
MRI: Magnetic resonance imaging
OP: Osteoporosis
OA: Osteoarthritis
PL: Posterolateral
STDB: Single-tunnel double-bundle
STSB: Single-tunnel single-bundle
SB: Single-bundle
SD: Standard deviation
